# Video-assisted thoracoscopic iodopovidone pleurodesis for malignant pleural effusions in moderate to high-risk Colombian patients

**DOI:** 10.34172/jcvtr.2020.52

**Published:** 2020-12-01

**Authors:** Juan Carlos Garzón, Eric Edward Vinck, Diana Carolina Cárdenas, Luis Jaime Téllez

**Affiliations:** ^1^Department of Thoracic Surgery and Lung Transplant, Fundación Cardioinfantil, Bogota, Colombia

**Keywords:** Malignant Pleural Effusions, Pleurodesis, Video-assisted Thoracoscopic Surgery, Iodopovidone-Iodine, LENT

## Abstract

***Introduction:*** In developing countries where talc may not be readily available, video-assisted thoracoscopic (VATS) iodopovidone pleurodesis offers an excellent alternative for the treatment of malignant pleural effusions (MPEs).

***Methods:*** This study analyzes a retrospective experience using VATS iodopovidone pleurodesis for malignant pleural effusions at a single cardiothoracic center in the capital of Colombia evaluating success according to LENT (Lactate, Eastern Cooperative Oncology Group-ECOG, Neutrophil-Lymphocyte ratio, Tumor type) scores. A total of 75 records of patients taken to VATS iodopovidone pleurodesis for MPEs were retrieved from our institutional database during a 5-year period from 2014-2019. Of these, 45 had complete clinical history data necessary to analyze both LENT scores and post-op follow-up imaging.

***Results:*** Of the 45 patients evaluated, 93.3% (42 patients) had either complete resolution of pleural effusions or partial resolution with an asymptomatic recovery within the first month post op. Chest pain was the most common postoperative complaint, which was present in 20% of patients. The mean postoperative ECOG score was 2±1.7. Patients with moderate to high-risk LENT scores had success rates of 96.7% and 92.3% respectively.

***Conclusion:*** Video-assisted thoracoscopic pleurodesis using Iodopovidone-iodine is an effective approach for MPEs. In developing countries where Iodopovidone iodine is readily available and affordable, patients may benefit from this agent with excellent results and minimal complications.

## Introduction

Malignant pleural effusions (MPEs) continue to be a therapeutic challenge for thoracic surgeons. MPEs universally indicate an advanced oncological stage and a life expectancy of five months causing significant patient discomfort and diminished quality of life.^1,2^ When malignant pleural effusions occur, their volume may progress until manifesting with respiratory symptoms and in rare cases present as a tension hydrothorax. ^1-3^ Both the diagnosis and treatment of malignant pleural effusions generally involve pleural drainage through thoracentesis. Up to 90% of MPEs reoccur requiring repeated drainage, at this point permanent catheters may be placed, or pleurodesis considered if there is adequate lung expansion.^2-5^ Contraindications for video-assisted thoracoscopic surgery (VATS) chemical pleurodesis include patient refusal, non-expanding lung, suspicion of current infection, concomitant adhesions requiring decortication, and less than one month of life expectancy. ^2,3^ Successful pleurodesis is defined as no re-accumulation for up to 30 days.^4,5^ Pleurodesis effectiveness or success can be classified as either complete, showing no effusion recurrence during follow-up chest x-ray; partial, resulting in minor asymptomatic recurrence during follow-up, or failed pleurodesis. Failed pleurodesis can be further sub-classified as primary, meaning continued and persistent effusion despite pleurodesis with impossibility to remove chest tubes, or secondary, resulting in symptomatic recurrence during follow-up.^2-5^ Since pleurodesis fails in up to 10%-40% of malignant pleural effusions, choosing the right patients for this procedure requires careful analysis. If pleurodesis fails or the patient has a trapped lung, intrapleural catheter placement becomes the treatment of choice.^2-6^

The two most used agents for chemical pleurodesis are talc and iodopovidone-iodine. In 1935 Bethune reported the first case of talc as a chemical agent for pleurodesis, and iodopovidone-iodine was first used twenty years later in 1955.^4-7^ Pleurodesis agents may be applied either through chest tubes or thoracoscopically, the former being the most studied. Although both thoracoscopic talc pleurodesis and iodopovidone-iodine chest tube pleurodesis have been evaluated thoroughly, VATS iodopovidone pleurodesis for MPEs has only been researched in a small number of series; the first was in 1991.^4-7^ Most studies involving VATS iodopovidone pleurodesis have been directed towards spontaneous and persistent pneumothorax, not MPEs. As a matter of fact, to our knowledge, the largest series reported, involved 40 patients taken to VATS iodopovidone-iodine pleurodesis for MPEs by Olivares-Torres et al with a success rate of 96%.^7^ In a small series by Sharshar et al in 2018, a 92.1% success rate was reported using medical thoracoscopy (pleuroscopy) iodopovidone-iodine pleurodesis for MPEs.^8^

The exact mechanism of action of iodopovidone in pleurodesis remains unclear; however proposed theories include enhanced pleural fibrosis related to low pH (pH, 2.97), production of fibroblast growth factor, and strong oxidative and cytotoxic properties of iodine inducing a potent inflammatory response. Iodopovidone also has anti-exudative properties which may be related to the chelation of proteins.^3-10^ Currently, thoracoscopic pleurodesis using iodopovidone-iodine for MPEs is an underused and under-research method.

In developing countries like Colombia, talc is not readily available; therefore, at an average cost of $3.30 per liter, iodopovidone-iodine offers an effective and affordable option for patients with MPEs. Despite many advances in general thoracic surgery in Colombia, the medical use of talc was not approved by the health ministry until 2018. ^11,12^ This study describes a retrospective analysis at a single cardiothoracic center in the capital of Colombia evaluating the effectiveness of VATS iodopovidone pleurodesis in MPEs according to LENT (Lactate dehydrogenase, Eastern Cooperative Oncology Group-ECOG, Neutrophil-Lymphocyte ratio, Tumor type) scores. Although the recently developed LENT score system evaluates survival primarily, it indirectly offers criteria for determining which patients would most likely benefit from pleurodesis and which should be offered a less invasive approach to their MPEs.^13,14^ Here we evaluate whether the LENT score may be applied in assessing pleurodesis success.

## Materials and Methods

This study represents a single-center retrospective descriptive cohort of patients taken to VATS pleurodesis. Institutional patient histories and databases were reviewed and evaluated. Ethics committee review and approval were attained for this study. Inclusion criteria included all patients presenting with MPEs confirmed by thoracentesis and fluid cytology reoccurring after initial or repeated needle drainage and taken to a VATS iodopovidone-iodine pleurodesis. A total of 75 patient records of patients taken to VATS iodopovidone pleurodesis for MPEs were retrieved from our institutional database during a 5-year period from 2014 to 2019. Of these, 45 had complete clinical history data necessary to analyze both LENT scores and post-op follow-up imaging; follow-up was conducted on an outpatient basis. Incomplete clinical history data, and failure to attend outpatient follow-up were the main reason for patient exclusion. We analyzed pre-op LENT and the post-operative ECOG scores, and follow-up chest x-rays of these patients in order to determine overall respiratory and general health improvement following surgery at 1 month post-operative. Successful VATS iodopovidone pleurodesis was defined as either complete radiographic resolution of pleural effusions or partial resolution with symptomatic improvement through post-op ECOG scores at one month following surgery. Table 1 outlines patient and pleural fluid characteristics. After signed informed consent, all patients were taken to a standard two-port VATS approach in a lateral decubitus position. A Fifth and Seventh intercostal space bi-portal approach in the anterior axillary line placement was used; after pleural effusion drainage and adequate lung expansion were confirmed, 120 mL of iodopovidone-iodine was sprayed in the pleural cavity intra-operatively. Single chest tubes were left in place and patients were carefully monitored for precipitated thyrotoxicosis in patients with subclinical hyperthyroidism (Jod-Basedow effect). Post-operative monitoring was done either in the intensive care unit or on the floors according to each patient´s needs. Continuous variables and Categorical variables were presented by mean ± standard deviation and frequencies with percentages, respectively.

## Results

A total of 45 patients were included in the final assessment. Of these, 28 (62.2%) were female patients; average age was 60.8±15.9 years. Primary tumor histologic distribution was pulmonary: 13 (28.8%), breast: 10 (22.2%), metastatic: 9 (20%), esophageal/gastric: 5 (11.1%), lymphoma: 4 (8.8%), gynecological: 3 (6.6%), mesothelioma: 1 (2.2%). The most frequent comorbidity was arterial hypertension in 14 patients (31.1%). Other comorbidities at a lesser degree included, hypothyroidism, deep vein thrombosis, diabetes, chronic obstructive pulmonary disease and others. Pre-surgical left ventricular ejection fraction (LVEF) was 57.5%±6.5. Pleural effusion was right-sided in 25 patients (55.5%), pleural fluid volume was 1171±635 mL, average lactate dehydrogenase (LDH) was 659 ±643 IU/L-^1^, and total fluid proteins 3.36 ±1.4 mg/dL. Serum neutrophil to leucocyte ratio averaged 9.76±20.5 and mean pre-op ECOG score was 3±0.9. All patients had chest tomographies as part of their workup and bronchoscopy was performed exclusively in patients with central or bronchial tumors or bronchial blockage on chest CT.

After data assessment patient distribution was: one patient (2.2%) with a low-risk LENT score, 31 patients (68.8%) with moderate-risk LENT scores, and 13 patients (28.8%) with High-risk LENT scores. These values yielded an average LENT score of 3.95±1.2 (moderate risk) for the study group (Table 1). In this study there were no intra-operative complications, one patient however presented with respiratory failure following surgery requiring intensive care admission. Perioperative complications included: two pleural fistulas, one surgical site bleeding, one pneumothorax, one patient had fever, one chylothorax, and another patient needed re-intervention due to adhesions. Post-operative pain was the main complaint following surgery in 9 patients (20%) and one patient died three weeks following surgery due to the natural course of her oncological pathology. Mean chest tube duration was 5 days, and post-op ECOG scores averaged 2±1.7 (Table 2).

**Table 1:** Patients and pleural fluid characteristics

Patients	45 (100%)
Age	60.8±15.9
Male	17 (37.7%)
Female	28 (62.2%)
Comorbidities	
Systemic hypertension Ave. LVEF%	14 (31.1%) 57.5%±6.5
Pleural fluid	
Pleural fluid volume, mL	1171±635
Left Right	20 (44.4%) 25 (55.5%)
Fluid LDH, IU/L-^1^	659 ±643
Fluid protein, mg/dL	3.36 ±1.4
Neutrophil/Leucocyte ratio	9.76±20.5
Malignancy	
Lung Breast Metastatic Esoph/Gastric Lymphoma Ginecological Mesothelioma	13 (28.8%) 10 (22.2%) 9 (20%) 5 (11.1%) 4 (8.8%) 3 (6.6%) 1 (2.2%)
Pre-op ECOG	3±0.9
Pre-op LENT score Low risk (0-1) Moderate risk (2-4) High risk (5-7) Average	1 (2.2%) 31 (68.8%) 13 (28.8%) 3.95±1.2
Total	45 (100%)

Abbreviations: LVEF, left ventricular ejection fraction; LDH, Lactate dehydrogenase; ECOG, Eastern Cooperative Oncology Group; LENT, Lactate, ECOG, Neutrophil-Lymphocyte ratio, Tumor type

**Table 2:** Study results and summary

	**No.**	**No pleural effusion** **(x-ray 1-month)**	**Improved ECOG w/ residual pleural effusion with (x-ray 1-month)**	**Success**
Pre-op LENT Score				
Low-risk	1 (2.2%)	0 (0%)	0 (0%)	0 (0%)
Moderate-risk	31 (68.8%)	15 (48.3%)	15 (48.3%)	30 (96.7%)
High-risk	13 (28.8%)	10 (76.9%)	2 (15.4%)	12 (92.3%)
Post-op ECOG score	2±1.7			
Days w/tube	5±2.4			
Peri-operative mortality	1 (2%)			
Post-op pain	9 (20%)			
Complications Pleural fistula Pneumothorax Chylothorax Fever Bleeding Respiratory failure Re-operation/Decortication Total	2 4%) 1 (2%) 1 (2%) 1 (2%) 1 (2%) 1 (2%) 1 (2%) 8 (17.7%)			
Total		25 (55.5%)	17 (37.7%)	42 (93.3%)

Abbreviations: ECOG, Eastern Cooperative Oncology Group; LENT, lactate, ECOG, neutrophil-lymphocyte ratio, tumor type

**Figure 1:**
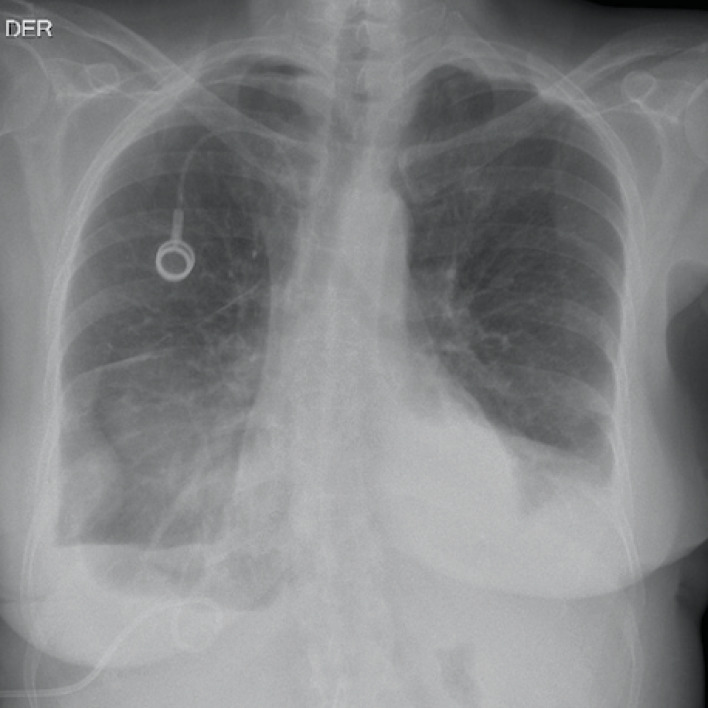
Example of a patient with an unsuccessful Left VATS pleurodesis

**Figure 2:**
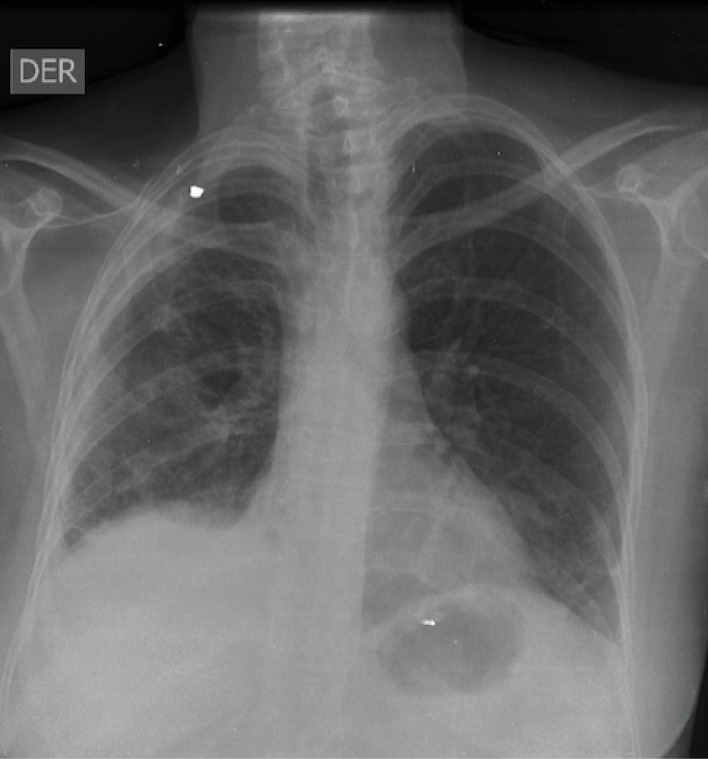
Example of a patient with a successful Right VATS pleurodesis

According to pre-op LENT score distribution, the single patient with a low-risk score had a failed pleurodesis requiring catheter placement; 0% success. In the Moderate-risk group of 31 patients (68.8%), 15 (48.3%) had no residual pleural effusions on follow-up chest x-rays and another 15 (48.3%) were asymptomatic with improved respiratory function despite residual pleural effusion on follow-up chest x-rays; a 96.7% success rate. Figures 1 and 2 illustrate examples of a patient with an unsuccessful pleurodesis and another with resolution following VATS. In the high-risk group of 13 patients (28.8%), 10 (76.9%) had complete resolution of pleural effusions on follow-up chest imaging and another 2 (15.4%) were asymptomatic with improved respiratory function despite residual pleural effusion on follow-up chest x-rays; 92.3% success rate. Table 2.

A total of 25 patients (55.5%) had complete resolution of pleural effusions on post-op imaging; complete success, along with symptomatic resolution. Asymptomatic patients having respiratory improvement with residual pleural effusions was 37.7% (17 patients); partial success. Total success rate was 93.3% (42 patients). Of the 25 patients with complete pleural effusion resolution, 36% (9 patients) were male and 64% (16 patients) were female. Follow-up was done on an out-patient basis at one month post-operative with chest x-rays. Because of the advanced oncological stage of the patients, mid- to long- term follow-up was not possible; this holds true to the palliative nature of Pleurodesis.

## Discussion

There is limited literature on the efficiency of VATS iodopovidone-iodine pleurodesis for MPEs.^5-11^ A 2016 Cochrane review consisting of 62 randomized trials including a total of 3 428 patients taken to pleurodesis yielded no studies involving a VATS iodopovidone-iodine pleurodesis approach for MPEs.^13^ Agarwal et al reported a meta-analysis evaluating N=157 patients with pleural effusions treated with iodopovidone pleurodesis; N=144 through chest tubes and N=121 through thoracoscopy (for pneumothorax). Success rates reached 95% with confidence intervals of 80-93%.^14,15^ The success rate of iodopovidone pleurodesis in recent studies was 90.6%, almost equal to the efficacy of talc pleurodesis (93%) and other agents used for chemical pleurodesis including silver nitrate (75%–90%) and quinacrine (64%–100%).^11-17^ However, none of these studies involved VATS iodopovidone for MPEs. Previous series show excellent outcomes (between 88.5% and 94.2% for MPEs) with iodopovidone pleurodesis through chest tubes.^9-15^ In one meta-analysis, Agarwal concluded that tube vs thoracoscopic iodopovidone pleurodesis yielded no significant differences in effectiveness for MPEs (success 88%).^14,15^

In this study, a success rate of 93.3% was seen using iodopovidone-iodine pleurodesis with a two-port VATS approach for MPEs. In geographical regions like Colombia with high prevelance rates of tuberculosis (TB), patients having significant pleural effusions refractory to needle drainage as well as non-conclusive fluid cytology, deserve an extra careful choice-making process when it comes to selecting the right chemical agent for pleurodesis. If patients with MPEs also have an undiagnosed pleural or lung TB, using talc for pleurodesis might be detrimental because of the risk of severe empyema. Patients however with a non-conclusive fluid cytology and a concomitant underlying TB taken to iodopovidone pleurodesis, have a reduced risk for empyema because of the antibiotic properties of iodopovidone-iodine.^11-15,17,18^ The most common complaints following iodopovidone pleurodesis include dyspnea, chest pain and fever. Previous series document an incidence of post-op chest pain of up to 48% for talc and 26% for iodopovidone, in a comparative study by Bakr and colleagues, iodopovidone had minor complications in comparison to other agents.^18-20^ In this series, post-op pain complaint was 20%, other minor complications were 17.7%.

During the last few years, the recently developed LENT score has allowed surgeons to stratify and predict survival/mortality; indirectly allowing better patient selection determining who will most likely fail pleurodesis with increased peri-operative mortality.^19,20^ A low-risk LENT score (0-1) indicates a 319 days life expectancy increasing the candidacy for pleurodesis. A moderate LENT score (2-4) predicts an overall life expectancy of 130 days reducing the chances of benefitting from pleurodesis, a high LENT score (5-7) indicates a life expectancy of 44 days making patients poor candidates for pleurodesis because of high perioperative mortality.^19,20^ Due to its recent introduction, the LENT score system has not yet been implemented as a standard in Latin America. Despite high-mortality and low-survival for high-risk LENT score patients, in this study, most patients 44 (97.7%) had moderate to high-risk LENT scores along with a 93.3% successful pleurodesis and improved respiratory function and symptoms. This study shows a success rate for VATS iodopovidone pleurodesis in MPEs paralleling other reports using iodopovidone through chest tubes and VATS using talc.

The retrospective nature and small patient sample size are the main limitations of this study. In addition, no formal statistical analysis was performed comparing preoperative variables of the LENT score.

## Conclusion

Video-assisted thoracoscopic pleurodesis using iodopovidone-iodine is an effective approach for MPEs. Although studies have shown the superiority of talc as the agent of choice in pleurodesis and silver nitrate as the optimal approach for previously failed pleurodesis, in developing countries where iodopovidone iodine is readily available and affordable, patients may benefit from this agent with excellent results and minimal complications.

## Acknowledgments

The authors would like to thank: Lina M. Ramírez; Nurse, Epidemiologist and Health Education Specialist for her assistance with data analysis.

## Competing interests

None.

## Ethical approval

This study was approved by the scientific research division and ethics committee of the Fundación Cardioinfantil, Bogota Institute. Approval to use/review patient histories was obtained from Fundación Cardioinfantil.

## Funding

None.

## References

[1] Trotter D, Aly A, Siu L, Knight S (2005). Video-assisted thoracoscopic (VATS) pleurodesis for malignant effusion: an Australian teaching hospital’s experience. Heart Lung Circ.

[2] Vinck EE, Garzón JC, Peterson T, Villarreal R, Cabrera L, Van den Eijnden L (2018). Tension hydrothorax: emergency decompression of a pleural cause of cardiac tamponade. Am J Emerg Med.

[3] Ibrahim IM, Dokhan AL, El-Sessy AA, Eltaweel MF (2015). Povidone-iodine pleurodesis versus talc pleurodesis in preventing recurrence of malignant pleural effusion. J Cardiothorac Surg.

[4] Mohsen TA, Abou Zeid AA, Meshref M, Tawfeek N, Redmond K, Ananiadou OG (2011). Local iodine pleurodesis versus thoracoscopic talc insufflation in recurrent malignant pleural effusion: a prospective randomized control trial. Eur J Cardiothorac Surg.

[5] Kahrom H, Aghajanzadeh M, Asgari MR, Kahrom M (2017). Efficacy and safety of povidone-iodine pleurodesis in malignant pleural effusions. Indian J Palliat Care.

[6] Echavarría A, Pinzón V, Barés JP, Fernández E (1991). [Intracavitary treatment of malignant pleural effusion with iodine-povidone]. Rev Med Panama.

[7] Olivares-Torres CA, Laniado-Laborín R, Chávez-García C, León-Gastelum C, Reyes-Escamilla A, Light RW (2002). Iodopovidone pleurodesis for recurrent pleural effusions. Chest.

[8] Sharshar RS (2018). Iodopovidone pleurodesis, diferent techniques, excellent efficacy in malignant pleural effusion Tanta University experience. Eur Respir J.

[9] Vinck EE, Martínez SI, Barrios RV, Téllez LJ, Garzón JC, García-Herreros L (2019). Facing the challenges of perioperative air leaks using water seal in Colombia. Asian Cardiovasc Thorac Ann.

[10] Vinck EE (2019). General thoracic surgery as a subspecialty in Colombia. J Thorac Cardiovasc Surg.

[11] Bibby AC, Dorn P, Psallidas I, Porcel JM, Janssen J, Froudarakis M (2018). ERS/EACTS statement on the management of malignant pleural effusions. Eur Respir J.

[12] Clive AO, Kahan BC, Hooper CE, Bhatnagar R, Morley AJ, Zahan-Evans N (2014). Predicting survival in malignant pleural effusion: development and validation of the LENT prognostic score. Thorax.

[13] Clive AO, Jones HE, Bhatnagar R, Preston NJ, Maskell N (2016). Interventions for the management of malignant pleural effusions: a network meta-analysis. Cochrane Database Syst Rev.

[14] Agarwal R, Aggarwal AN, Gupta D, Jindal SK (2006). Efficacy and safety of iodopovidone in chemical pleurodesis: a meta-analysis of observational studies. Respir Med.

[15] Agarwal R, Khan A, Aggarwal AN, Gupta D (2012). Efficacy & safety of iodopovidone pleurodesis: a systematic review & meta-analysis. Indian J Med Res.

[16] Godazandeh G, Haji Qasemi N, Saghafi M, Mortazian M, Tayebi P (2013). Pleurodesis with povidone-iodine, as an effective procedure in management of patients with malignant pleural effusion. J Thorac Dis.

[17] Aelony Y (2003). Talc pleurodesis vs iodopovidone. Chest.

[18] Das SK, Saha SK, Das A, Halder AK, Banerjee SN, Chakraborty M (2008). A study of comparison of efficacy and safety of talc and povidone iodine for pleurodesis of malignant pleural effusions. J Indian Med Assoc.

[19] Bakr RM, El-Mahalawy II, Abdel-Aal GA, Mabrouk AA, Ali AA (2012). Pleurodesis using different agents in malignant pleural effusion. Egypt J Chest Dis Tuberc.

[20] Makkar A, Patni S, Joad AK, Lakhera KK (2017). An observational study on safety and efficacy of povidone-iodine for pleurodesis in cancer patients. South Asian J Cancer.

